# Draft Genome Sequence of the Extremely Haloalkaliphilic and Homoacetic Bacterium Natroniella acetigena Z-7937^T^

**DOI:** 10.1128/mra.00472-22

**Published:** 2022-08-11

**Authors:** Yan Huang, Masaru K. Nobu, Kensuke Igarashi, Souichiro Kato

**Affiliations:** a Graduate School of Agriculture, Hokkaido University, Sapporo, Japan; b Bioproduction Research Institute, National Institute of Advanced Industrial Science and Technology, Sapporo, Japan; c Bioproduction Research Institute, National Institute of Advanced Industrial Science and Technology, Tsukuba, Japan; Montana State University

## Abstract

Natroniella acetigena Z-7937^T^ (= DSM 9952^T^) is a heterotrophic homoacetogenic natronophile. The draft genome sequence is 2.6 Mb in 116 contigs, with a G+C content of 34.1%.

## ANNOUNCEMENT

Acetogens are found in nature worldwide, and over 100 species have been discovered (1). Given their ability to fix carbon, acetogens play vital roles in the biogeochemical carbon cycle. Natroniella acetigena Z-7937^T^ is a mesophilic, haloalkaliphilic acetogen growing at high salinity of up to 26% and pH of up to 10.7 and requiring sodium carbonate and chloride ions (2). The haloalkaliphile was isolated from the bottom mud of soda-depositing Lake Magadi and is capable of using lactate, ethanol, pyruvate, glutamate, and propanol, with acetate as the primary end product (2). However, unlike its halophilic counterparts Acetohalobium arabaticum and Fuchsiella alkaliacetigena of the order *Haloanaerobiales*, N. acetigena Z-7937^T^ was not able to grow chemolithoautotrophically with H_2_/CO_2_, although findings were positive for the original isolate (2–6). Despite its uniqueness as a functional member in soda lake ecology (7), little is known about N. acetigena Z-7937^T^ with respect to metabolism mechanism, physiological function, and ecological niche. Here, we present the draft genome of N. acetigena Z-7937^T^.

Strain Z-7937^T^ was obtained from the culture collection DSMZ and cultivated anaerobically in DSMZ medium 784. Genomic DNA was extracted from cells at log phase by using the MasterPure Gram-positive DNA purification kit (Epicentre) according to the manufacturer’s instructions. Draft genome sequencing was performed by using the Illumina HiSeq 2000 platform (100-bp paired-end reads) at Hokkaido System Science (Hokkaido, Japan). The paired-end library was constructed by using the TruSeq DNA PCR-free library preparation kit (Illumina). A total of 4.53 Gb of paired-end raw reads was generated and trimmed by fastp v0.20.1 (–detect_adapter_for_pe –W 6 –M 20 –r –l 78) (8). The clean data were sequentially assembled by using SPAdes v3.14.1 (–isolate –k 21, 33, 41, 65, 77) (9). The assembly resulted in a 2.6-Mb draft genome containing 116 contigs (>1,000 bp). The genome coverage was 350×. The G+C content was 34.1% as calculated by BBmap v38.18 (default parameters) (https://sourceforge.net/projects/bbmap). Genome annotation was conducted by the automated NCBI Prokaryotic Genome Annotation Pipeline (PGAP) v6.1 (10). This genome contains 2,558 genes, including 2,499 protein-coding genes, 5 rRNA genes (5S, 16S, and 23S), 50 tRNA genes, and 4 noncoding RNA genes. The genome annotation was double-checked by NCBI CD-Search (11). The annotation suggested that the genome contained all genes encoding enzymes of the Wood-Ljungdahl pathway. Genes encoding hydrogenases and the Rnf complex were also present, supporting the hypothesis that N. acetigena conserves energy via electron transfer phosphorylation based on an ATPase-involving chemiosmotic mechanism (12). Further comparative genomic analyses will provide a better understanding of the metabolism and adaptation strategies of haloalkaliphilic acetogenic bacteria in high salinity and pH.

### Data availability.

This draft genome and the raw sequence data have been deposited in NCBI GenBank and the Sequence Read Archive (SRA) under the accession numbers JALKBX000000000 and SRR19259016, respectively.
